# Differential NF-κB mRNA Expression in Blood and Buccal Mucosa of Pediatric Patients with RSV Bronchiolitis

**DOI:** 10.3390/genes16080851

**Published:** 2025-07-22

**Authors:** Francesco Savino, Cristina Calvi, Stefano Gambarino, Maddalena Dini, Anna Pau, Paola Montanari, Anna Clemente, Ilaria Galliano, Massimiliano Bergallo

**Affiliations:** 1 Early Infancy Special Care Unit, Regina Margherita Children Hospital, A.O.U. Città della Salute e della Scienza di Torino, 10126 Turin, Italy; francesco.savino@unito.it; 2 Pediatric Laboratory, Department of Public Health and Pediatric Sciences, University of Turin, Regina Margherita Children’s Hospital, 10126 Turin, Italy; cristina.calvi@unito.it (C.C.); stefano.gambarino@unito.it (S.G.); maddalena.dini@unito.it (M.D.); anna.pau@unito.it (A.P.); paola.montanari@unito.it (P.M.); anna.clemente@unito.it (A.C.); massimiliano.bergallo@unito.it (M.B.)

**Keywords:** RSV, bronchiolitis, NF-κB, buccal swab, peripheral blood

## Abstract

Background: Respiratory syncytial virus (RSV) bronchiolitis is a leading cause of lower respiratory tract infections in children under two years of age. NF-κB is a key transcription factor in antiviral and inflammatory responses. This study investigates the expression of NF-κB mRNA in both blood and buccal swab samples of pediatric patients hospitalized for RSV bronchiolitis, comparing levels at admission and discharge. Methods: Paired peripheral blood and buccal swab samples were collected from pediatric patients (*n* = 85) at hospital admission and discharge. Quantitative real-time PCR was used to assess NF-κB mRNA levels. Results: NF-κB mRNA levels significantly decreased in blood between admission and discharge (*p* < 0.05), while no significant change was observed in buccal swabs. Conclusions: These results suggest a compartment-specific regulation of NF-κB, with systemic inflammatory resolution at discharge and persistent or distinct mucosal immune activity. Understanding these dynamics may improve our approach to monitoring and treating RSV bronchiolitis.

## 1. Introduction

Respiratory syncytial virus (RSV) is the most common viral agent causing bronchiolitis and lower respiratory tract infections in infants and young children, frequently resulting in hospitalization, especially during the first year of life [1]. Despite the typically self-limiting nature of RSV infections, a significant subset of patients develops severe inflammation and airway obstruction, requiring supportive care [2]. The host immune response, particularly the balance between pro- and anti-inflammatory mediators, plays a critical role in determining disease severity and outcome [3].

Among the key regulators of the inflammatory cascade is nuclear factor kappa-light-chain-enhancer of activated B cells (NF-κB), a transcription factor complex that governs the expression of numerous genes involved in immune responses, including cytokines, chemokines, adhesion molecules, and acute-phase proteins [4,5]. NF-κB activation has been implicated in the pathogenesis of several respiratory viral infections, including RSV, influenza, and coronaviruses [6]. In the context of RSV, NF-κB signaling has been shown to be activated both in airway epithelial cells and in circulating immune cells, contributing to the production of inflammatory mediators such as IL-6, TNF-α, and IL-8 [7,8].

Previous studies have demonstrated increased NF-κB activity in the respiratory tract during the acute phase of RSV infection [9], but little is known about the temporal and compartment-specific regulation of NF-κB during the course of the disease. While peripheral blood samples are often used to assess systemic immune responses, they may not reflect the local mucosal immune activation occurring in the respiratory epithelium. Conversely, buccal swabs—non-invasive and easy to obtain—can provide access to epithelial RNA and potentially serve as a surrogate for respiratory mucosal sampling [10].

The aim of the present study was to evaluate NF-κB mRNA expression levels in both blood and buccal swabs collected from pediatric patients with confirmed RSV bronchiolitis at the time of hospital admission (representing the acute phase) and at discharge (representing clinical recovery). We hypothesized that NF-κB expression would decrease upon recovery, reflecting the resolution of inflammation, and sought to determine whether this trend would be observed equally in systemic (blood) and local (buccal mucosa) compartments.

## 2. Materials and Methods

### 2.1. Study Population

The study included patients admitted to the “Neonatal and Infant Pathology” Department of the “Regina Margherita” Children’s Hospital in Turin during the RSV epidemic from December 2023 to April 2024. The study population was restricted to patients who tested positive for RSV on admission, resulting in a sample size of 85 individuals. Within the pediatric sample analyzed (40 males and 45 females), no significant differences were found in terms of gender distribution. The mean age was 63.25 days, with 12 patients being preterm (born under 37 weeks’ gestation) and 15 patients having been previously or subsequently admitted to a neonatal intensive care unit. In terms of the type of oxygen therapy required, 50 patients received HFNC, 3 received C-PAP, and 1 received bubble C-PAP, reflecting the complexity of the cases in terms of the need for high-flow oxygen therapy.

On admission to the ward (or the next morning if admission was after 3 p.m.), a buccal swab and a blood sample were taken from all patients and sent to laboratories for analysis. The same procedure was repeated 24 h after ventilator weaning or at the expected time of discharge.

### 2.2. Sample Storage

A total of 200 μL of whole blood was added to 800 µL of RNApro solution (BioMole, Turin, Italy) in a 1.5 mL Eppendorf tube and resuspended by vortexing [11]. Buccal swabs were resuspended in 1 mL of RNApro solution (Biomole, Turin, Italy) in a 1.5 mL Eppendorf tube and vortexed. Samples were stored at −80 °C.

### 2.3. Total RNA Extraction

Total RNA was extracted from whole blood and from buccal swabs using the Maxwell automated extractor (Promega, Madison, WI, USA). The RNA extraction process includes DNase treatment. RNA concentration and purity were determined by conventional UV spectrophotometry, using the NanoDrop (Thermo Fisher Scientific, Waltham, MA, USA). The RNA concentration of each sample was within the accepted range. The A260/A280 ratio was used to determine RNA purity, with a ratio of 1.8–2.1 indicating highly purified RNA. RNA extracts were amplified directly without reverse transcription to exclude contamination with genomic DNA and stored at −80 °C until use.

### 2.4. Reverse Transcription

Four hundred nanograms of total RNA were reverse-transcribed with 2 μL of buffer 10×, 4.8 μL of MgCl_2_ 25 mM, 2 μL ImpromII (Promega), 1 μL of RNase inhibitor 20 U/L, 0.4 μL random hexamers 250 μM (Promega), 2 μL mix dNTPs 100 mM (Promega), and dd-water in a final volume of 20 μL. The reaction mix was carried out in a GeneAmp polymerase chain reaction (PCR) system 9700 Thermal Cycle (Applied Biosystems, Waltham, MA, USA) under the following conditions: 5 min at 25 °C, 60 min at 42 °C, and 15 min at 70 °C for the inactivation of enzyme; the cDNAs were stored at −80° until use.

### 2.5. Evaluation of Co-Infections

Nasal swabs, collected at admission, were analyzed for the detection of additional viral agents besides RSV. The detected viral agents included SARS-CoV-2, identified with an antigenic test, and Rhinovirus, Coronavirus OC43, Coronavirus HK, Coronavirus NL63, Coronavirus 229E, Metapneumovirus, Influenza virus A and B, Parainfluenza virus 1, 2, and 3, tested using Real-Time PCR.

Sets of primers and probes for all detected viruses were designed using Primer Express Software Version 3.0 (Thermo Fisher, Waltham, MA, USA). The primers/probes concentrations were 300/150 nM for each target. Real-Time PCR assays were performed using the GoTaq qPCR Master Mix (Promega, Milano, Italy), on the 7500 Real-Time PCR System (Life Technologies Ltd., Carlsbad, CA, USA) instrument. Reactions were performed in a 96-well plate with the following thermal profile: 95 °C for 10 min, followed by 40 cycles at 95 °C for 10 s and 60 °C for 30 s. Amplification was set in a final volume of 20 μL, including 4 μL of cDNA.

### 2.6. Transcription Levels of NF-κB by RT-PCR

H18S was selected as the reference gene in all determinations due to its stability as a reference gene. Relative Quantification of mRNA concentrations for NF-κB was performed using the ABI PRISM 7500 real-time system (Thermofisher Scientific, Waltham, MA, USA).

Fifty ng of RNA were reverse transcribed and amplified using the primer previously reported [12], and Taqman probe FAM-CAGGCAGCCTCCAGCCCAGTGA-BHQ1 new design with Primer Express Software (ThermoFisher) in a reaction with a total volume of 20 μL.

Reactions were performed in a 96-well plate with the following thermal profile: 50 °C for 10 min, 95 °C for 10 min, followed by 40 cycles at 95 °C for 10 s and 60 °C for 30 s. Each sample was run in triplicate. Relative Quantification of target gene transcripts was performed using the ΔΔCt method [13]. Consequently, expression changes were calculated, and results were expressed in corresponding arbitrary units, known as Relative Quantification (RQ).

All analyses were conducted in a Biosafety Level 2 (BSL-2) laboratory, following NIH [14] guidelines.

### 2.7. Statistical Analysis

The Mann–Whitney test was used to compare transcription levels of NF-κB at admission and at discharge in buccal swabs and blood. The Spearman correlation test assessed correlations between mRNA levels of NF-κB between blood and mucosal samples. Statistical analyses were conducted using Prism software, Version 7 (GraphPad Software, La Jolla, CA, USA). A *p*-value < 0.05 was considered statistically significant.

## 3. Results

### 3.1. Co-Infections

Additional viral agents besides RSV were detected in nasal swabs collected upon admission to the ward. Eight samples tested positive for co-infections: three for Coronavirus HK, one for Coronavirus OC43, one for Coronavirus 229E, one for Rhinovirus, one for SARS-CoV-2, and one for Influenza A.

### 3.2. NF-κB Expression

NF-κB expression was evaluated for bronchiolitis patients at admission and discharge. Results were expressed in arbitrary units, called RQ (Relative Quantification).

**Figure 1:**
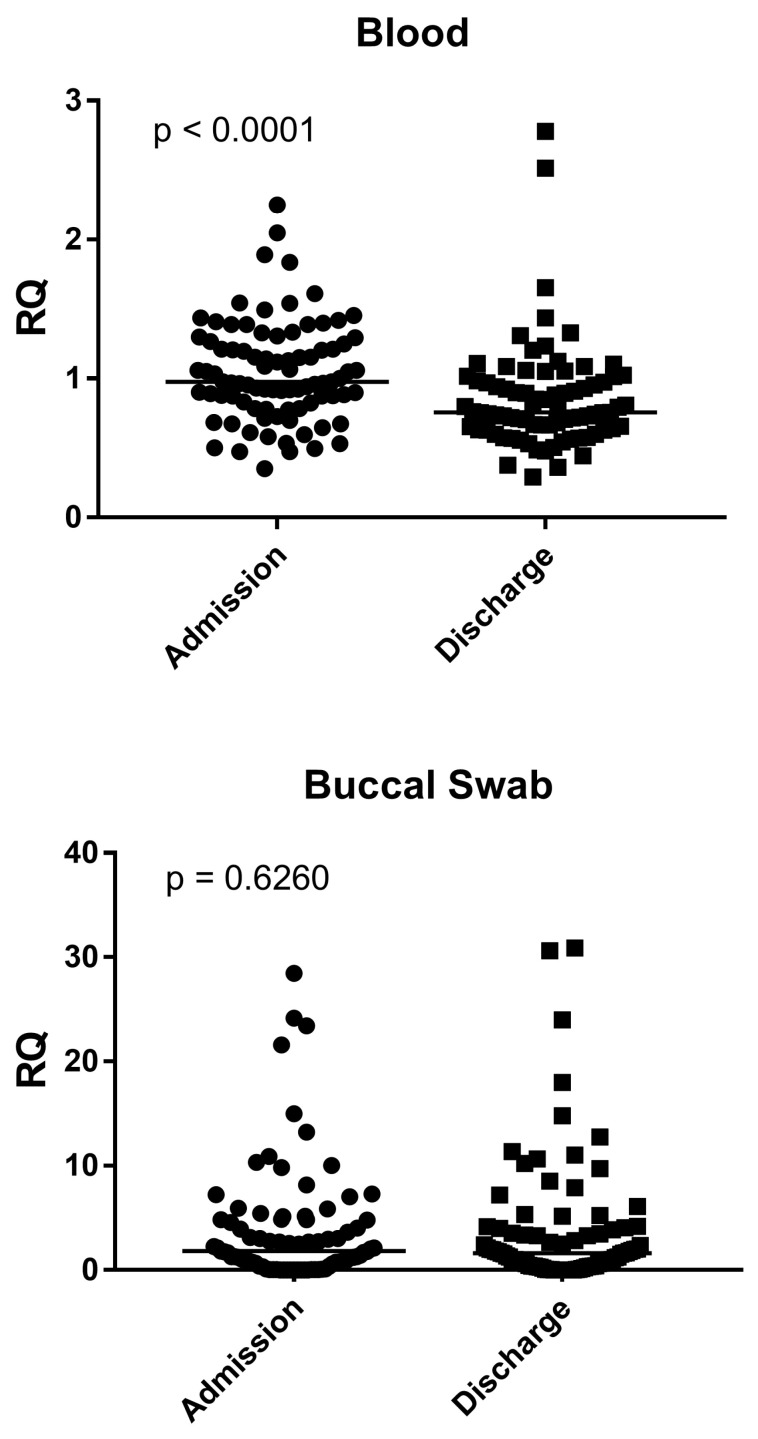
Expression of NF-κB in whole blood and buccal swabs from children with RSV Bronchiolitis at admission and discharge. RQ = Relative Quantification; Admission: RSV-positive bronchiolitis patients at admission; Discharge: RSV-positive bronchiolitis patients at discharge; Circles and squares show the median of three individual measurements, horizontal lines the median values.

As reported in Figure 1, in blood samples, the NF-κB expression was significantly higher in bronchiolitis patients at admission than at discharge (*p* < 0.0001). In buccal swabs, the NF-κB expression in bronchiolitis patients at admission and at discharge was the same (*p* = 0.6260) (Figure 1).

The medians and IQR 25–75% were: Blood: bronchiolitis at admission 0.98, 0.83–1.27; bronchiolitis at discharge 0.76, 0.64–1.00; Nasal swab: bronchiolitis at admission 1.80, 0.53–4.80; bronchiolitis at discharge 1.62, 0.37–3.89.

### 3.3. Correlation Between Blood and Mucosal Samples

**Figure 2:**
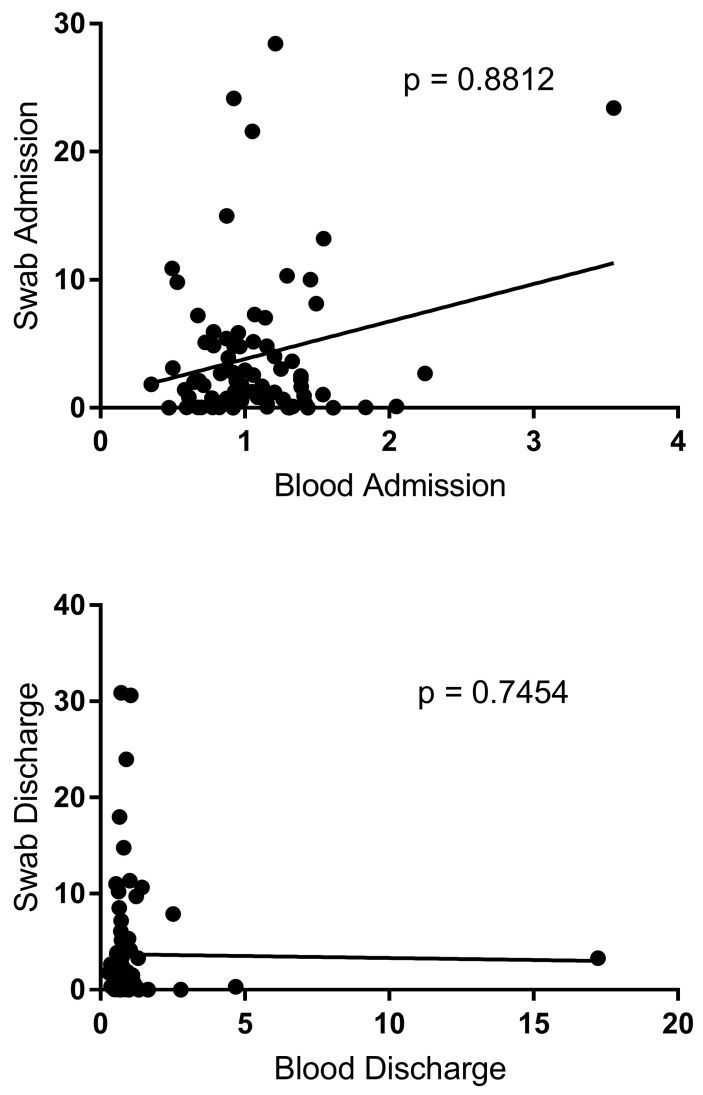
Correlation between blood and buccal swab in bronchiolitis patients at admission and at discharge. Circles show the mean of three individual measurements. Line: linear regression line.

No significant correlation was found between NF-κB expression of blood and NF-κB expression of buccal swabs in bronchiolitis children at admission and discharge, *p* = 0.8812 and *p* = 0.7454, respectively (Figure 2).

## 4. Discussion

This study demonstrates a statistically significant decrease in NF-κB mRNA expression in the peripheral blood of pediatric patients hospitalized with RSV bronchiolitis from admission to discharge, while no such difference was observed in buccal swab samples. These findings suggest that systemic inflammation, as reflected by NF-κB transcriptional activity, resolves during the clinical course of RSV bronchiolitis, whereas local mucosal NF-κB expression remains relatively unchanged. The observed systemic reduction aligns with the previous literature showing the resolution of inflammatory markers such as IL-6, TNF-α, and C-reactive protein during recovery from RSV [15]. NF-κB is a key upstream regulator of these cytokines, and its decreased expression in blood may reflect reduced transcriptional demand once the acute viral phase resolves. This decline could be attributed to the restoration of immune homeostasis and downregulation of circulating innate immune cells, such as monocytes and neutrophils that express NF-κB [16].

The stable expression in buccal mucosa raises interesting considerations. Firstly, buccal epithelial cells may retain a certain degree of NF-κB activation beyond the resolution of clinical symptoms due to prolonged exposure to viral antigens or epithelial remodelling [17]. The mucosal surface of the airway is the second largest surface area in the human body and serves as the primary defense against respiratory pathogens, including viruses [18]. This barrier consists of structural cells (such as epithelial, endothelial, and mesenchymal cells) immersed in an active viscoelastic gel matrix known as airway mucus [19]. Nasopharyngeal samples from children infected with RSV frequently exhibit an IFN response signature, even though most patients do not show detectable levels of secreted IFNs at the transcriptional level. These observations suggest that the nasal mucosa may not be the main source of secreted IFNs during these infections [16]. RSV is known to weakly induce interferon production within the respiratory tract. In infants infected with RSV, there is increased activation of IFN-related genes in peripheral blood samples 4 to 6 weeks post-infection compared to the acute phase, indicating potential suppression of the IFN response during the acute RSV infection phase [18]. A similar mechanism might be relevant for NF-κB, as demonstrated in our study. Alternatively, the local mucosal immune response may follow a different kinetic pattern, with delayed or sustained activation compared to systemic compartments. This is consistent with the findings from studies on mucosal immunity in RSV, where epithelial cells continue expressing immune genes even after viral clearance [18,20].

Another consideration is tissue-specific regulation. Buccal swab samples primarily contain keratinized epithelial cells, whereas blood samples represent a heterogeneous population of leukocytes. These differences in cellular composition inherently affect gene expression profiles. For example, epithelial NF-κB activation may be modulated by epigenetic mechanisms, local microbiota interactions, or sustained exposure to damage-associated molecular patterns (DAMPs) [21].

The need for minimally invasive biomarkers to monitor the course of RSV infection in very young infants is critical due to the challenges associated with obtaining systemic samples such as blood. Buccal swabs represent a practical and less distressing alternative, enabling easier repeated sampling in this fragile population. Our findings highlight the complexity of using surrogate sampling (such as buccal swabs) to monitor systemic disease processes. While buccal swabs offer a non-invasive and practical alternative for pediatric populations, they may not adequately reflect changes in systemic inflammation. This suggests that careful consideration must be given to the selection of sampling sites in biomarker-based studies, especially in pediatric populations where invasive sampling is limited.

While our findings indicate that NF-κB mRNA expression in buccal cells does not parallel the systemic inflammatory resolution, assessing gene expression in these cells may still provide valuable information on local mucosal immune status. Such data can complement systemic markers and may help identify subclinical mucosal inflammation or remodeling processes that persist beyond clinical recovery. Ultimately, this approach could guide more targeted follow-up and intervention strategies, improving outcomes in pediatric respiratory infections.

Clinically, the decline in blood NF-κB mRNA could potentially serve as a biomarker of recovery in RSV bronchiolitis, although further validation in larger cohorts is needed. Additionally, the persistence of NF-κB expression in buccal samples might provide insight into residual or subclinical mucosal inflammation, potentially relevant for long-term outcomes such as post-bronchiolitis wheezing.

A limitation of this study is the lack of age-matched healthy controls, which would be essential to better determine whether the observed buccal gene expression changes are infection-specific. However, recruiting a sufficient number of healthy neonates and young infants poses considerable ethical and logistical challenges. Future studies should include healthy controls to strengthen the comparison.

We also did not quantify NF-κB protein levels or correlate mRNA expression with cytokine profiles, which would offer a more comprehensive view of immune activity. Future studies should aim to include healthy controls to strengthen the comparison and to integrate multi-omic approaches, including transcriptomic, proteomic, and epigenetic profiling, across multiple anatomical compartments.

In conclusion, our study reveals a significant decrease in NF-κB mRNA expression in blood—but not buccal swabs—between admission and discharge in pediatric patients with RSV bronchiolitis. This compartmentalized pattern of immune regulation underscores the importance of sample origin in immunological studies and raises important questions about local versus systemic immune resolution in viral infections. Further studies are warranted to explore the clinical utility of NF-κB as a biomarker and to better understand the implications of persistent mucosal immune activity following RSV infection.

## Data Availability

The data supporting this study are available at an aggregate or population level upon reasonable request to the corresponding author.

## References

[1] Heemskerk S., van Heuvel L., Asey T., Bangert M., Kramer R., Paget J., van Summeren J. (2024). Disease burden of RSV infections and bronchiolitis in young children (<5 years) in primary care and emergency departments: A systematic literature review. Influenza Other Respir. Viruses.

[2] Meissner H.C. (2016). Viral bronchiolitis in children. N. Engl. J. Med..

[3] Collins P.L., Melero J.A. (2011). Progress in understanding and controlling respiratory syncytial virus: Still crazy after all these years. Virus Res..

[4] Hayden M.S., Ghosh S. (2011). NF-κB in immunobiology. Cell Res..

[5] Hoffmann A., Baltimore D. (2006). Circuitry of NF-κB signaling. Immunol. Rev..

[6] Haeberle H.A., Takizawa R., Casola A., Brasier A.R., Dieterich H.J., Van Rooijen N., Gatalica Z., Garofalo R.P. (2002). Respiratory syncytial virus-induced activation of nuclear factor-kappaB in the lung involves alveolar macrophages and toll-like receptor 4-dependent pathways. Infect. Dis..

[7] Bao X., Indukuri H., Liu T., Liao S.L., Tian B., Brasier A.R., Garofalo R.P., Casola A. (2010). IKKε modulates RSV-induced NF-κB-dependent gene transcription. Virology.

[8] Gambadauro A., Galletta F., Li Pomi A., Manti S., Piedimonte G. (2024). Immune response to respiratory viral infections. Int. J. Mol. Sci..

[9] Pickles R.J., Chen G., Randell S.H. (2024). Enhanced susceptibility of pediatric airway epithelium to respiratory syncytial virus infection. J. Clin. Investig..

[10] Zimmermann B.G., Park N.J., Wong D.T. (2007). Genomic targets in saliva. Ann. N. Y. Acad. Sci..

[11] Gambarino S., Galliano I., Clemente A., Calvi C., Montanari P., Pau A., Dini M., Bergallo M. (2024). Characteristics of RNA stabilizer RNApro for peripheral blood collection. Diagnostics.

[12] Chancharoenthana W., Kamolratanakul S., Udompornpitak K., Wannigama D.L., Schultz M.J., Leelahavanichkul A. (2025). Alcohol-induced gut permeability defect through dysbiosis and enterocytic mitochondrial interference causing pro-inflammatory macrophages in a dose dependent manner. Sci. Rep..

[13] Livak K.J., Schmittgen T.D. (2001). Analysis of relative gene expression data using real-time quantitative PCR and the 2^−ΔΔCT^ method. Methods.

[14] National Institutes of Health (NIH) (2024). NIH Guidelines for Research Involving Recombinant or Synthetic Nucleic Acid Molecules (NIH Guidelines).

[15] Hall C.B., Weinberg G.A., Iwane M.K., Blumkin A.K., Edwards K.M., Staat M.A., Auinger P., Griffin M.R., Poehling K.A., Erdman D. (2009). The burden of RSV in young children. N. Engl. J. Med..

[16] Stenvinkel P., Ketteler M., Johnson R.J., Lindholm B., Pecoits-Filho R., Riella M., Heimbürger O., Cederholm T., Girndt M. (2005). IL-10, IL-6, and TNF-alpha: Central factors in the altered cytokine network of uremia--the good, the bad, and the ugly. Kidney Int..

[17] Barnes M.V.C., Openshaw P.J.M., Thwaites R.S. (2022). Mucosal immune responses to respiratory syncytial virus. Cells.

[18] da Silva R.P., Thomé B.L., da Souza A.P.D. (2023). Exploring the immune response against RSV and SARS-CoV-2 infection in children. Biology.

[19] Ardain A., Marakalala M.J., Leslie A. (2020). Tissue-resident innate immunity in the lung. Immunology.

[20] Openshaw P.J., Tregoning J.S. (2005). Immune responses and disease enhancement during respiratory syncytial virus infection. Clin. Microbiol. Rev..

[21] Glaser L., Coulter P.J., Shields M., Touzelet O., Power U.F., Broadbent L. (2019). Airway epithelial derived cytokines and chemokines and their role in the immune response to respiratory syncytial virus infection. Pathogens.

